# Risk Factors for Mercury Exposure of Children in a Rural Mining Town in Northern Chile

**DOI:** 10.1371/journal.pone.0079756

**Published:** 2013-11-20

**Authors:** Johan Ohlander, Stella Maria Huber, Michael Schomaker, Christian Heumann, Rudolf Schierl, Bernhard Michalke, Oskar G. Jenni, Jon Caflisch, Daniel Moraga Muñoz, Ondine S. von Ehrenstein, Katja Radon

**Affiliations:** 1 Center for International Health Ludwig-Maximilians-University (LMU) at the Institute and Outpatient Clinic for Occupational, Social and Environmental Medicine, University Hospital Munich (LMU), Munich, Germany; 2 Department for Statistics, Ludwig-Maximilians-University Munich, Munich, Germany; 3 Institute for Ecological Chemistry, Helmholtz Center Munich - German Research Center for Environmental Health, Neuherberg, Germany; 4 Department of Pediatrics, University Children’s Hospital Zürich, Zürich, Switzerland; 5 Facultad de Medicina, Universidad Diego Portales, Santiago, Chile. Past staff of Facultad de Medicina, Latin American Center of the Center for International Health Ludwig-Maximilians-University, Universidad Católica del Norte, Coquimbo, Chile; 6 Department of Community Health Sciences, University of California Los Angeles, Los Angeles, California, United States of America; Indian Institute of Toxicology Reserach, India

## Abstract

**Objective:**

Traditional gold mining is associated with mercury exposure. Especially vulnerable to its neurotoxic effects is the developing nervous system of a child. We aimed to investigate risk factors of mercury exposure among children in a rural mining town in Chile.

**Methods:**

Using a validated questionnaire distributed to the parents of the children, a priori mercury risk factors, potential exposure pathways and demographics of the children were obtained. Mercury levels were measured through analyzing fingernail samples. Logistic regression modeling the effect of risk factors on mercury levels above the 75^th^ percentile were made, adjusted for potential confounders.

**Results:**

The 288 children had a mean age of 9.6 years (SD = 1.9). The mean mercury level in the study population was 0.13 µg/g (SD 0.11, median 0.10, range 0.001–0.86 µg/g). The strongest risk factor for children’s odds of high mercury levels (>75^th^ percentile, 0.165 µg/g) was to play inside a house where a family member worked with mercury (OR adjusted 3.49 95% CI 1.23–9.89). Additionally, children whose parents worked in industrial gold mining had higher odds of high mercury levels than children whose parents worked in industrial copper mining or outside mining activities.

**Conclusion:**

Mercury exposure through small-scale gold mining might affect children in their home environments. These results may further help to convince the local population of banning mercury burning inside the households.

## Introduction

Since 1900, Chile’s history and economy has been highly influenced by its wealth of mineral resources [1]. In 2009, the export of noble metals like copper and gold still amounted to 57% of Chile’s total export [2]. At the same time, possible health hazards caused by toxic by-products of mining operations is a controversial topic in Chile [3].

In Chile, artisanal mining is practiced by so-called “Pirquineros”: small-scale miners (men or women, however rarely children) who descend into narrow pits, provisionally supported by wooden slats, to mine and to bring up ore-bearing stone. The stone is then ground to powder and mixed with liquid mercury (Hg), creating gold amalgam. This compound is heated over a Bunsen burner; the Hg evaporates and is thus separated from the pure gold as a result of this process [4], [5]. As this so-called “amalgamation” is often performed inside the private houses or patios of the miners, the miner himself and all individuals in the vicinity are exposed to the Hg vapors. The Hg vapors are thereafter effectively taken up by the blood stream and can further easily pass the blood-brain barrier to accumulate particularly in the cerebellum where it can affect protein synthesis, destroy membranes, and denature intracellular and cytoskeleton proteins, and enzymes [6]. Subsequent neurological abnormalities have already been documented among gold miners [7]. However, the adverse effects may be especially harmful for the developing organism of a child [4], [8]–[13].

The site of this study was a small rural town of 10,000 inhabitants in Region IV. at 1053 m above sea level. There are numerous small-scale gold mines and gold shops in town where the workers excavate the gold in the described traditional way as well as two large industrial gold and copper mines, located within the town limits. As parts of the community are concerned about potential adverse effects of the mining activities for the health and the well-being of the population, the Universidad Católica del Norte was asked by a community group and local politicians to investigate potential health effects in children.

The objective of this study was to investigate risk factors of Hg exposure of children due to small-scale mining. If such associations are found, intervention strategies should be put into place.

## Methods

### Ethics Statement

The study was approved by the Ethics Committee of the University Hospital Munich (LMU) (Project-No. 399-08) and by the Ethics Committee of the University Católica del Norte in Coquimbo, Chile (Project-No. 04/08). Written informed consent was obtained from the legal guardians of the children and from the children themselves.

The principles of the Declaration of Helsinki with its amendment of Somerset West, 1996, were considered in each part of the study.

### Study population

The study population of this cross-sectional population-based study consisted of schoolchildren attending grades 1 to 6 in the two major public schools of the town. We focused on these two public schools particularly for the following reasons:

1. The schools had a potentially high participation of our target group: children of small-scale gold miners.
2. The schools were the only schools in the community offering a complete elementary education.
3. 83.5% of children in schools offering education from grades 1 to 6 were registered at these two schools.

### Study Design and Procedure

The data used for this paper originated from two sources:

- Questionnaires to assess socio-demographic characteristics, parental occupation and general environmental information
- Fingernail sampling to measure Hg exposure

### Questionnaire

The questionnaire consisted of 52 items (table 1) mainly assembled from 5 validated questionnaires in Spanish language [14]–[20]. The questionnaire covered potential exposure pathways, risk factors, demographics and potential confounders for Hg exposure.

**Table 1:** Questionnaire Items.

Outcome	Reference	Item
Demographic and biometricdata:	Estudio Internacional sobre la saludrespiratoria en escolares [16] 1	sex, age, height, weight, number of siblings
	Encuesta Mundial de SaludEscolar [19] 2	birth year, marital state of parents
	Encuesta de exposición ambientala plomo en niños [17] 3	housing situation
Duration and intensity ofHg exposure:	Encuesta de exposición ambientala plomo en niños [17]	Domestic circumstances: birthplace of the child, domiciles and frequency/time spent inside the house
	Global Mercury Project – Environmental andHealth Assessment [18]	Professional situation of the parents: profession, time of employment, Hg-contact of the mother during pregnancy, individuals in the household working with Hg
	Global Mercury Project – Environmental andHealth Assessment [18]	Whereabouts of the child: time spent in school/most common playground (inside/outside the house)
Predictors of Hgexposure:	Global Mercury Project – Environmental andHealth Assessment [18]	fish consumption

1 International Study on Asthma and Allergies in Childhood (ISAAC).

2 Global School-based Student Health Survey (GSHS).

3 Questionnaire of environmental lead-exposure in children.

To assess the feasibility of the questionnaire, it was tested among ten volunteers with a similar socioeconomic background as the study participants. The pilot group was first asked to complete the questionnaire, to explain some of the questions to the investigators and to comment on comprehensibility; this led to small modifications of the questionnaire to improve understanding.

### Fingernail Sampling and Mercury Analysis

Long-term Hg exposure was measured by examining fingernails of the children. This method was used because of its simple and non-invasive way of sampling, and the stability and easy handling of the samples. One study identified fingernails as the best discriminator of Hg-exposure in dentists [21] and finger and toenail samples constitute reliable markers of long term Hg exposure [21]–[23].

Participating children were asked to not cut their fingernails 3 weeks prior to the study. Before sampling, short-term exogenous contamination was eliminated by washing the children’s nails with soap and bottled water. The cutting was performed with a customary stainless steel nail clipper. The samples were stored in Eppendorf tubes, labeled with the respective study ID and sent to Germany for analysis.

Samples were weighed into quartz vessels and were subjected to digestion with 1 ml HNO_3_. The vessels then underwent high-pressure digestion for ten hours at 170°C. After adding ultrapure water (18.5 MOhm) up to the 10 ml mark, total-Hg was analyzed by inductivity coupled plasma-sector field mass spectrometry (ICP-sf-MS) [24], [25]. For quality control, every 10^th^ measurement, 3 matrix blank determinations and a control determination of a standard for Hg were included and analyzed together with the samples. The final results were calculated using a computerized lab-data management system that related the measurements to calibration curves, blanks, control standards and the sample-weights. The parents were informed about the respective Hg concentrations of their children and recommendations to limit the Hg exposure were given to families and local authorities.

### Statistical Analysis

Due to a partially low item non-response, multiple imputations (MI) of missing values were made using the R package Amelia II (version 1.6–3). MI is based on the assumption that the variables are multivariate normally distributed and missing at random (MAR) [26]. As the Hg values were not normally distributed, they were Box-Cox transformed before imputation [27]. The probability of the MAR assumption was increased through including in the MI also variables not intended for the final analysis [26]. The imputation resulted in 7 complete data sets.

All statistical analyses were made with both the incomplete and the imputed data. Descriptives for the imputed data were calculated using Rubin’s rules [28]. Using the R package Zelig (version 3.5–5), bivariate and multivariate logistic regression models adjusted for potential confounders were made on the association between a priori risk factors and Hg values above the 75^th^ percentile. A priori risk factors considered were: gender, fish consumption (>4 times per week, 1–4 times per week versus <1 time per week), fathers work place (industrial copper mine, traditional gold mining, outside mining versus industrial gold mine), mothers contact with Hg (yes versus no) and the collapsed variable ‘somebody working with Hg in household AND child mainly playing indoors’ (yes versus no). As no reference values for Hg levels in fingernails exist, we used the 75th percentile as a cut off; this method ensured sufficient power in the regression models. According to recommendations [29], the cut-off was chosen prior to analysis. All analyses were made using R Statistical Software version 2.15-1 and SPSS 20.

## Results

Of the 418 eligible school children, the parents of 288 children completed the questionnaire (response 69%) (table 2). Of these, 32.6% were complete cases, 32.3% had 1 missing value, 14.9% had 2 missing values and 20.1% had ≥3 missing values (data not shown). Overall, pre and post imputation descriptives were similar (table 3). Before imputation, children’s mean age was 9.6 years (SD = 1.9) and 54% were male. Approximately every third participant had a mother who reported having been in contact with Hg during pregnancy. Moreover, approximately every fifth child ate fish more than 4 times per week. Overall, 40.5% of working fathers were miners of which 18.8% were artisanal miners.

**Table 2:** Questionnaire and fingernail sampling response among children of the two major public schools of the town.

Test	Comment	Participation n (%)
Population:	total	432 (100.0)
	drop-outs^1^	14 (3.2)
	study population	418 (96.8)
Questionnaire:	handed-out	418 (100.0)
	returned	338 (80.9)
	at least partially completed	288 (68.9)
Fingernail sampling:		219 (52.4)

1 Children who dropped out at various points in time because of relocation or absence for other reasons than health.

**Table 3:** Pre and post imputation descriptives of all study variables.

Variable	Pre imputation		Post imputation (N = 288)^1^		NA^2^ (%)
	Mean (SD)	N (%)	Mean (SD)	N (%)	
Age^3^ (years)	9.59 (1.93)		9.59 (1.92)		0
Hg (µg/g)	0.13 (0.11)		0.13 (0.10)		36.1
Sex:					0
Male		156 (54.2)		156 (54.2)	
Female		132 (45.8)		132 (45.8)	
Mother in contact withHg during pregnancy:					12.5
No		176 (69.8)		197 (68.4)	
Yes		76 (30.2)		91 (31.6)	
Father’s occupation^3^:					18.8
Industrial gold mine		9 (3.8)		19 (6.6)	
Industrial copper mine		42 (17.9)		54 (18.9)	
Traditional gold mining		44 (18.8)		55 (19.0)	
Outside mining		139 (59.4)		160 (55.6)	
Hg exposure in householdand child playing inside:					17.7
No		211 (89.0)		253 (87.8)	
Yes		26 (11.0)		35 (12.2)	
Fish consumption:					4.2
<1 times/week		85 (30.8)		89 (30.9)	
1–4 times/week		131 (47.5)		136 (47.2)	
>4 times/week		60 (21.74)		63 (21.9)	
Number of siblings^3^:					5.9
0		26 (9.6)		29 (10.1)	
1–2		170 (62.7)		176 (61.1)	
>2		75 (27.7)		83 (28.8)	
Hours spent indoors^3^:					22.2
<3 hours/day		23 (10.3)		33 (11.8)	
3–6 hours/day		49 (21.9)		67 (26.7)	
>6 hours/day		152 (67.8)		189 (61.5)	
Mother employed^3^:					8.3
No		191 (72.4)		209 (72.6)	
Yes		73 (27.6)		79 (27.4)	
Father employed^3^:					13.5
No		23 (9.2)		30 (10.4)	
Yes		226 (90.8)		258 (89.6)	
Somebody smoking in household^3^:					10.1
No		188 (72.6)		209 (72.6)	
Yes		71 (27.4)		79 (27.4)	

1 Descriptives for variables post imputation were calculated using Rubin’s rules.

2 NA = missing value. Column displays percentage of missing values in variable.

3 Variable additionally included in imputation model to improve missing at random assumption.

The imputation yielded an increase of fathers working in industrial gold mines (6.6% vs. 3.8%) and a slight decrease of children spending their time indoors >6 hours per day (61.5% vs. 67.8%) (table 3).

The Hg-levels were also similar when comparing pre and post imputation estimates. The pre-imputation descriptives showed among the 219 children whose fingernail samples were obtained (table 2) a mean Hg-level of 0.13 µg/g (SD 0.11, median 0.10 µg/g, range: 0.001 to 0.86 µg/g) (table 3) (median and range not shown in table). This Hg-level was consistent with that yielded post imputation (table 3). Post imputation, a total of 69 children had Hg-levels above the 75th percentile (0.165 µg/g) (table 4).

**Table 4:** Main risk factors for mercury exposure above the 75th percentile (0.165 μg/g).

Risk factor		Pre imputation^1^	Post imputation^2^	
	N (%) >75th Hg-percentile	Adjusted OR 95% CI	Unadjusted OR 95% CI	Adjusted OR 95% CI
Sex:				
Male	42 (26.9)	1	1	1
Female	27 (20.5)	0.46 (0.19–1.10)	0.69 (0.37–1.28)	0.60 (0.30–1.19)
Fish consumption:				
<1 times/week	15 (16.9)	1	1	1
1–4 times/week	36 (26.5)	0.96 (0.36–2.57)	1.58 (0.76–3.29)	1.57 (0.74–3.26)
>4 times/week	18 (28.6)	1.16 (0.34–4.01)	0.79 (0.48–1.29)	0.78 (0.46–1.32)
Father working in:				
Industrial gold mine	5 (26.3)	1	1	1
Industrial copper mine	10 (18.5)	0.51 (0.07–3.92)	0.58 (0.12–2.79)	0.52 (0.10–2.58)
Traditional gold mining	15 (27.3)	0.80 (0.11–5.91)	0.95 (0.22–4.20)	0.78 (0.17–3.51)
Outside mining	39 (24.4)	0.71 (0.12–4.18)	0.83 (0.19–3.71)	0.82 (0.19–3.51)
Mother in contact with Hg during pregnancy:				
No	43 (21.8)	1	1	1
Yes	25 (27.7)	1.10 (0.41–2.91)	1.35 (0.62–2.94)	1.03 (0.46–2.31)
Hg work in household and child playing inside:				
No	52 (20.6)	1	1	1
Yes	17 (48.5)	4.91 (1.41–17.03)	3.40 (1.28–9.07)	3.49 (1.23–9.89)

Descriptive data, pre imputation (adjusted) and post imputation (unadjusted and adjusted) logistic regression models with odds ratios (OR) and 95% confidence intervals (95% CI). N = 288.

1 Pre imputation adjusted odds ratios (for all variables in table).

2 Post imputation unadjusted and adjusted (for all variables in table) odds ratios based on all seven imputed datasets combined.

In both the bivariate and multivariate models, children whose parents worked with industrial gold mining showed higher odds of being exposed to Hg, compared to children whose parents worked in industrial copper mining, traditional gold mining or were outside of mining (table 4). Moreover, females had a lower risk of Hg exposure than males. The strongest risk factor for Hg exposure above the 75^th^ percentile was, both in the unadjusted and adjusted model, ‘mainly playing inside a household where somebody was working with Hg’ (OR adjusted: 3.49 95% CI 1.23–9.89).

## Discussion

This paper describes one of the very few studies in the field of Hg exposure in children. Our results indicate relevant Hg exposure levels in fingernails of children, especially when they are in close contact with traditional gold mining. The median fingernail Hg level in this study was 0.10 µg/g with a broad range. We are unaware of any validated reference values and the number of earlier studies using fingernail samples is limited. However, similar median levels, albeit with higher maximum values, have been found in earlier studies. Rees et al. found a median concentration of 0.16 µg/g Hg (range = 0.04–1.15 µg/g) in nails [30] in a randomly selected population of 27 individuals exposed to Hg through fish consumption. A Nicaraguan study investigating Hg exposure in children working in gold mines also found a median level of 0.16 µg/g ranging up to 2.72 µg/g [23].

Our risk models showed a statistically significantly higher risk of having elevated levels of Hg in fingernail samples (OR 3.49 95% CI 1.23–9.89) for children who frequently play in environments where artisanal gold extraction activities are performed. This result could be an important finding with regard to prevention of children’s Hg exposure. As we adjusted this association for children’s fish consumption, organic Hg exposure should not have been a confounder. Instead, this result indicates relevant environmental exposure of children whose parents are occupationally exposed to Hg.

Study strengths include the uniqueness of the study, the relatively representative sample, and the overall high response. The last can partly be traced back to the strong support of stakeholders in the community involved, such as the town mayor, community groups, principals, teachers, and staff of the schools. Moreover, through involving the two biggest public schools in town, easy access to a representative and heterogeneous study group including children whose families carry out artisanal mining was ensured.

As Hg use was based on parental self-report it is possible that the use of Hg in the living environment was occasionally under-reported. This might have led to a misclassification of exposure and weakened associations between investigated risk factors and fingernail Hg levels. Moreover, as children in private schools were not included, the generalizability of found exposure levels is possibly limited. Further, the quality of fingernails as long-term markers of Hg exposure might be a limitation of our Hg assessment. However, a number of studies have shown that fingernail samples can be used to measure long-term Hg exposure if exposure is constant, which can be assumed in our study population [21]–[23]. Finally, we cannot rule out bias of our Hg levels from amalgam in children’s teeth.

Our analysis was based on MI, which today is a standard approach in epidemiology as the loss of power and the error introduced by excluding cases with missing values is considered a major bias [31]. Using MI, we increased our relatively low sample size, resulting in a higher statistical power. Our pre and post imputation risk estimates differed, however not much. The observed difference is explained by the difference in statistical power and by the different assumptions the complete case (CC) respective the MI analysis requires met. In fact, the more frequently used CC-analysis requires more assumptions met to yield unbiased estimates than does the MI (MCAR respective MAR). Although the MAR assumption cannot be tested for [32], it is the most common situation in epidemiological research [33]. Thus, the imputed estimates should be more reliable than those yielded by the CC analysis.

Our paper shows how community initiated research can be carried out successfully through involving the local community, local stakeholders as well as schools, and local and foreign universities. Having reported our results, the community decided to appoint places in a certain distance to the village where the Hg burning will take place from now on. The authorities as well as the families were both highly interested in further cooperation and the possibility of an intervention.

## Conclusions

Our findings suggest that children in a rural mining area with small-scale mining activities in Chile are at risk for chronic Hg exposure. The identification of such setting specific exposure pathways may help to develop and target measures to reduce children’s Hg exposure from occupational sources. As a next step, associations between Hg exposure and the children’s health outcomes will be evaluated.
