# Correction: Risk Factors for Mercury Exposure of Children in a Rural Mining Town in Northern Chile

**DOI:** 10.1371/journal.pone.0144527

**Published:** 2015-12-02

**Authors:** Johan Ohlander, Stella Maria Huber, Michael Schomaker, Christian Heumann, Rudolf Schierl, Bernhard Michalke, Oskar G. Jenni, Jon Caflisch, Daniel Moraga Muñoz, Ondine S. von Ehrenstein, Katja Radon

The Hg-values of n = 5 children analyzed by the authors were recorded with an incorrect unit, and should instead have been 1000 times higher. The authors have now updated all results based on the new, correct values. The authors apologize for this error and confirm that the main results remain when calculated based on the correct Hg-values.

Please see an updated version of Table 4 here, which displays the final adjusted associations.

**Table 4:** Main risk factors for mercury exposure above the 75th percentile (0.177 μg/g). Descriptive data, pre imputation (adjusted) and post imputation (unadjusted and adjusted) logistic regression models with odds ratios (OR) and 95% confidence intervals (95% CI). N = 288.

Risk factor		Pre imputation^1^	Post imputation^2^	
	N (%) > 75th Hg-percentile	Adjusted OR 95% CI	Unadjusted OR 95% CI	Adjusted OR 95% CI
Sex:				
Male	43 (27.6)	1	1	1
Female	29 (22.0)	0.71 (0.36–1.38)	0.73 (0.36–1.49)	0.74 (0.38–1.46)
Fish consumption:				
<1 times/week	19 (21.3)	1	1	1
1–4 times/week	37 (27.2)	1.62 (0.71–3.70)	1.18 (0.59–2.33)	1.15 (0.59–2.23)
>4 times/week	16 (25.4)	1.25 (0.45–3.49)	0.81 (0.47–1.40)	0.68 (0.40–1.14)
Father working in:				
Industrial gold mine	5 (26.3)	1	1	1
Industrial copper mine	10 (18.5)	0.65 (0.10–4.23)	0.45 (0.07–2.86)	0.52 (0.08–3.13)
Traditional gold mining	20 (36.3)	2.50 (0.40–15.75)	1.31 (0.23–7.53)	2.05 (0.39–10.70)
Outside mining	37 (23.1)	0.70 (0.13–3.91)	0.68 (0.14–3.29)	0.93 (0.23–3.73)
Mother in contact with Hg during pregnancy:				
No	41 (20.8)	1	1	1
Yes	31 (34.1)	1.68 (0.83–3.43)	1.92 (1.06–3.48)	1.08 (0.47–2.46)
Hg work in household and child playing inside:				
No	55 (21.7)	1	1	1
Yes	17 (48.6)	5.55 (1.98–15.56)	3.11 (1.35–7.19)	3.32 (1.26–8.73)

^1^ Pre imputation adjusted odds ratios (for all variables in table).

^2^ Post imputation unadjusted and adjusted (for all variables in table) odds ratios based on all seven imputed datasets combined.

The main changes include:

- A slightly smaller OR of the main association between 'Hg work in household and child playing inside' and Hg-values > the 75th percentile (new OR = 3.32, 95% CI 1.26–8.73) vs. (old OR = 3.49, 95% CI 1.23–9.89).
- Traditional gold mining is now a risk factor of having Hg-values > the 75th percentile, when compared with industrial gold mining (OR = 2.05, 95% CI 0.39–10.70).
- The updated Hg-value corresponding to the 75th percentile is 0.177 ug/g (old value = 0.165 ug/g).
